# Physiological Performance Curves: When Are They Useful?

**DOI:** 10.3389/fphys.2021.805102

**Published:** 2021-12-02

**Authors:** Alexander G. Little, Frank Seebacher

**Affiliations:** ^1^Department of Biology, Biosciences Complex, Queen’s University, Kingston, ON, Canada; ^2^School of Life and Environmental Sciences, University of Sydney, Darlington, NSW, Australia

**Keywords:** reaction norm, adaptation, developmental plasticity, reversible acclimation, climate change, environment

## Abstract

This review serves as an introduction to a special issue of Frontiers in Physiology, focused on the importance of physiological performance curves across phylogenetic and functional boundaries. Biologists have used performance curves to describe the effects of changing environmental conditions on animal physiology since the late 1800s (at least). Animal physiologists have studied performance curves extensively over the past decades, and there is a good foundation to understanding how the environment affects physiological functions of individuals. Our goal here was to build upon this research and address outstanding questions regarding the mutability and applicability of performance curves across taxonomic groups and levels of biological organization. Performance curves are not fixed at a taxonomic, population, or individual level – rather they are dynamic and can shift in response to evolutionary pressures (e.g., selection) and epigenetic programming (e.g., plasticity). The mechanisms underlying these shifts are being increasingly used to predict the efficacy with which plasticity and heritability of performance curves can render individuals and populations less vulnerable to climate change. Individual differences in physiological performance curves (and plasticity of performance curves) can also have cascading effects at higher levels of biological organization. For instance, individual physiology likely influences group behaviors in non-additive ways. There is a need therefore to extend the concept of performance curves to social interactions and sociality. Collectively, this special issue emphasizes the power of how within- and between-individual shifts in performance curves might scale up to the population-, species-, and community-level dynamics that inform conservation management strategies.

## Introduction

Physiological performance curves describe changes in physiological rates with respect to an environmental gradient. The y-axis can represent any level of physiological performance, from single-molecule (e.g., enzyme kinetics) to whole-animal measures (e.g., locomotion, metabolic rate, and reproductive output). Similarly, the x-axis can describe a wide range of environmental gradients, including those associated with abiotic (e.g., temperature, salinity, and oxygen-concentration) and biotic factors (e.g., infection, competition, and predation). The shapes of physiological performance curves thereby describe physiological rates as a function of the acute environment. The precise shapes can vary within and between taxonomic and functional boundaries. Determining the mechanisms that drive these changes is important for understanding the diverse strategies and limitations that shape animal responses to their environments.

A comparative approach has been particularly powerful to help trace the cascading effects of environmental change across levels of biological organization. Reductionist approaches focusing on single-enzyme thermal performance curves, for instance, indicate that breakdowns in animal performance at high temperatures cannot simply be ascribed to protein denaturation, as was once thought. Rather, the shapes of these single-enzyme curves suggest that decreases in whole-animal performance likely reflect more nuanced shifts in enzyme microstates and activation energies (see Schulte, 2015). Thus, understanding how lower levels of performance change across environmental gradients can signal important consequences for emergent traits at higher levels of organization. Thermal sensitivity of heart rate scope, for instance, can indicate whole-animal and population-level performance, such as migration success in Pacific salmon (Eliason et al., 2011).

In an even broader sense, a comparative approach has also helped identify the adaptive and plastic (i.e., developmental plasticity and reversible acclimation) responses that animals use to overcome environmental variability. By comparing performance curves *between* populations and species, for instance, differences in shape can point to adaptations that promote fitness in local environments. Comparing how performance curves shift as a function of the environment *within* individuals and populations, on the other hand, can indicate whether plasticity allows animals to defend performance against environmental change. These comparative approaches can also help to assess tradeoffs associated with specific physiological strategies.

In this Special Issue, we focus on molecular and physiological mechanisms underlying performance curves and how performance curves may shift in response to an environmental change, by both genetic and epigenetic mechanisms. Responses to the environment are likely to differ between phylogenetic groups, and we are particularly interested in differences between ectotherms and endotherms. Along these lines, we also aim to extend the concept of performance curves from individuals to groups of animals to ask how the physiology of performance curves of individuals can impact emergent properties, like social behavior.

### History of Physiological Performance Curves

In the simplest terms, physiological performance curves effectively represent dose-response relationships – an increasing “dose” of some environmental factor alters the response of a physiological system. Many ancient societies clearly recognized the phenomenon of dose-dependence. Ancient Greeks, for instance, recognized that wine in excess would dull the senses (e.g., Homer’s Odyssey; Papakonstantinou, 2009), and herbal remedies in Ancient China were recommended to be doubled daily, then increased 10-fold if a cure was not yet achieved (Waddell, 2010). It was Paraclesus (c. 16th century), however, who synthesized our modern appreciation for dose-response relationships by stating that “all things are poison, and nothing is without poison; the dosage alone makes it so a thing is not a poison” (see Waddell, 2010 for review). Paraclesus was originally generous in his definition of a poison, even including compounds like oxygen and water. It is therefore little reach to extrapolate his model to include grades of other environmental factors, including temperature, UV-radiation, or even social stress.

**Figure 1:**
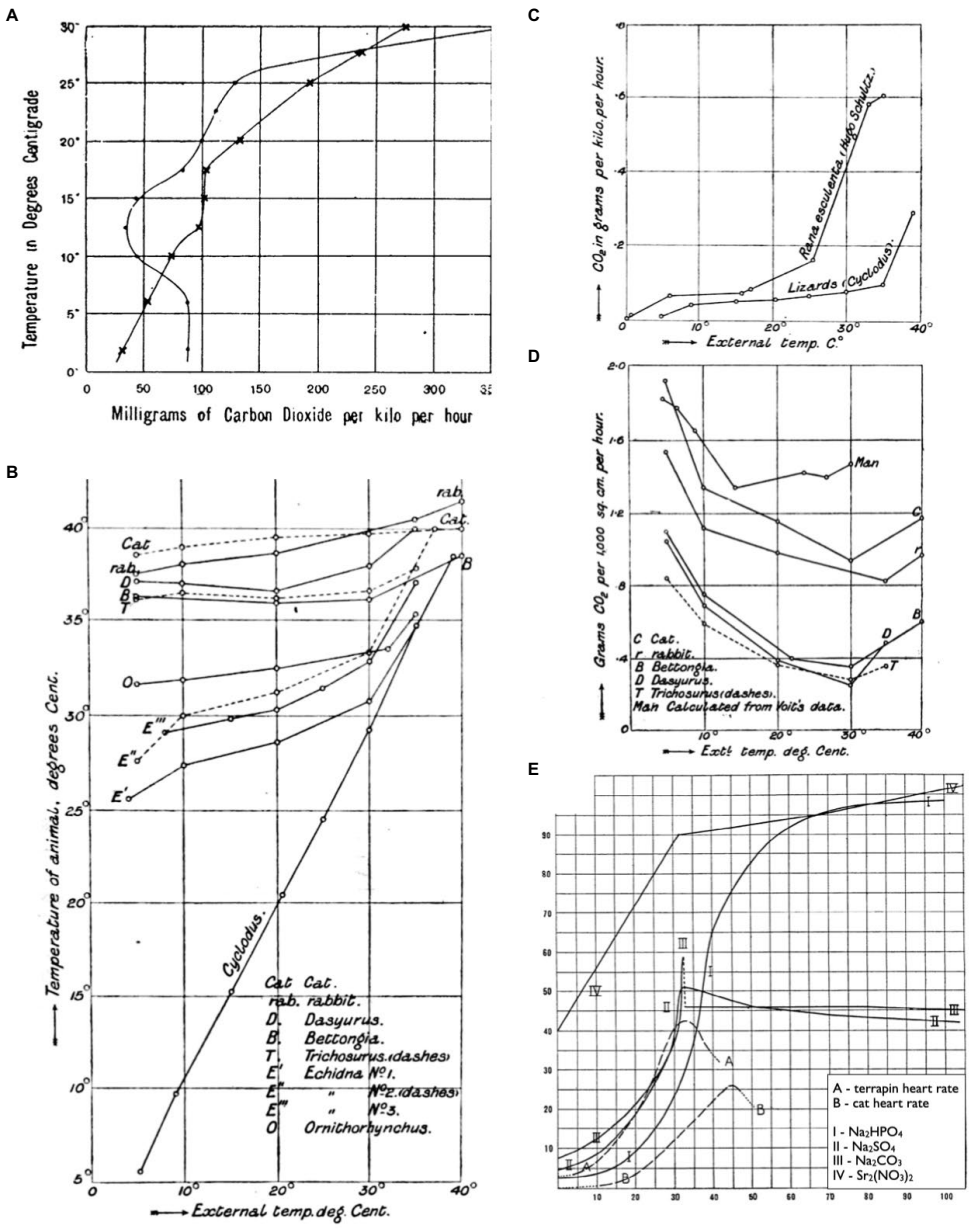
Thermal reaction norms as early examples of performance curves. CO_2_-production of a frog warmed from 2 to 32°C, then cooled back down to 2°C again the following day (**A**; reprinted from Vernon, 1894). Body temperatures of thermoregulators and thermoconformers respond differently to changes in ambient temperature (**B**; reprinted from Martin, 1903). CO_2_-production of thermoconformers **(C)** and thermoregulators **(D)** reveal that different metabolic properties underlie each evolutionary strategy (reprinted from Martin, 1903). Thermal sensitivities of heart rates for terrapin ventricle and a cat heart alongside thermal solubility curves for Na_2_HPO_4_, Na_2_SO_4_, Na_2_CO_3_, and Sr_2_(NO_3_)_2_ (**E**; adapted from Snyder, 1908).

Some of the first ecologically focused physiological performance curves came in the form of thermal performance curves in the late 19th century (Figure 1). Vernon (1894), for instance, plotted the thermal sensitivity of metabolism (as CO_2_-production) in a frog warmed from 2 to 32°C, then cooled back down to 2°C again the following day (Figure 1A). Martin (1903) plotted body temperatures of various animals as a function of ambient temperature to separate animals that thermoconformed (ectotherms) from those that thermoregulated (endotherms; Figure 1B). He subsequently plotted thermal performance curves for metabolic rate (CO_2_-production) between these various taxonomic groups to investigate the metabolic properties that correspond to these distinct evolutionary strategies (Figures 1C,D). Later, Snyder (1908) plotted terrapin and cat heart rates over a thermal gradient to investigate the basis of dynamic changes in their thermal sensitivities. This work was particularly novel in that Snyder compared the shapes of these curves with solubility curves for various physiological salts (e.g., Na_2_CO_3_; Figure 1E) in an attempt to identify the mechanisms that limit performance at high temperatures. While his hypothesis on “ion-proteid” interference was subsequently deemed incorrect, his approach may have been the first to compare such curves down levels of organization in search of a mechanistic explanation.

Since these early experiments on thermal sensitivity, physiological performance curves have been used to describe animal responses to a wide array of environmental factors. These types of curves have been useful not only in understanding animal responses to environmental stress (i.e., conformers vs. regulators), but also understanding the physiological strategies and limitations that bound these responses. The critical oxygen tension (*P*_crit_), for instance, represents the lowest PO_2_ at which an oxyregulator can regulate some rate of metabolic oxygen consumption (MO_2_), below which it oxyconforms (Tang, 1933). Comparing inflection points in performance at lower (e.g., hemoglobin-binding) and higher (e.g., locomotion) levels of organization has garnered great insights for mechanisms contributing to oxyregulation (e.g., McBryan et al., 2013).

### Performance: A Caveat

The concept of performance is to some degree subjective and does not necessarily correlate well with measures of fitness. During development, for instance, elevated resting metabolic rates (RMR) may promote higher fitness by implying increased rates of growth and development (Burton et al., 2011). In other cases, however, increased RMRs may reflect lower fitness as a consequence of reductions in aerobic scope (the oxygen use available for fitness-related activities; see McKenzie, 2011 for review). It is therefore important to consider specific contexts when assigning meaning to measures of performance. The disconnect between performance and fitness can be especially apparent when performance is measured at lower levels of biological organization, which are further removed from the emergent traits on which selection acts. An increase in the maximum activity of a single enzyme may mean very little unless it represents an important regulatory step in its broader biochemical pathway. Even seemingly direct links between lower-level processes and animal fitness can be obscured by tradeoffs within and between levels of organization. Such tradeoffs may mean that performance fails to track with fitness in intuitive ways. Thus, an important caveat is that high levels of performance at one level of organization do not necessarily translate to high levels of performance at another. It is also important to recognize that without empirical support, which is quite rare, it is very difficult to link measures of performance, even at the whole-animal level, with fitness outcomes in animal systems.

## The Curves

### Curve Shapes

**Figure 2:**
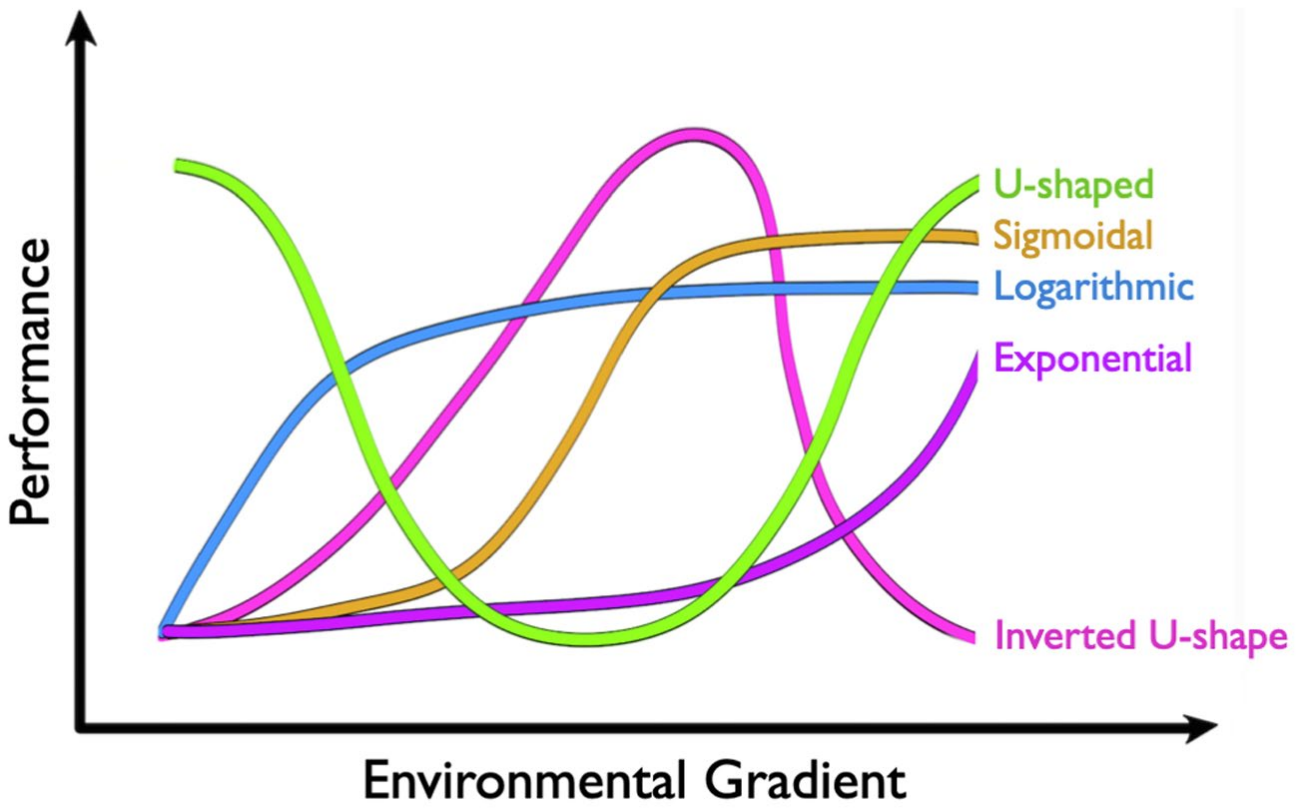
Common shapes of physiological performance curves include U-shape, sigmoidal, logarithmic, exponential, and inverted U-shape.

Performance curves take a number of shapes that can vary greatly across environmental and physiological contexts (Figure 2). The *inverted U-shape* arguably represents the most common shape for a physiological performance curve, where performance is maximized at some intermediate environmental state and decreases as the environment changes in either direction. Thermodynamic effects on enzymatic rate processes, for instance, mean that catalytic activity is often optimized at some temperature that may be reflective of local environmental conditions. Cooler temperatures reduce performance by limiting the free energy available for activation, whereas warm temperatures ultimately limit performance by shifting the distribution of active enzyme microstates and potentially raising activation energies (see Schulte, 2015). The shapes of these thermal performance curves also tend to be mirrored at higher levels of biological organization, like muscle function (e.g., Rall and Woledge, 1990), aerobic scope (see McKenzie, 2011 for review), and locomotor performance (e.g., Brett, 1967). Some curves, on the other hand, may appear to be more *U-shaped*. However, these curves tend to describe rates that are inversely proportional to performance. RMRs in marine osmoregulators, for instance, tend to be lowest in isosmotic conditions and increase as salinity changes in either direction. While the subsequent curve is invariably U-shaped, rising RMRs reflect metabolic costs of osmoregulation, which ultimately act as loading factors on aerobic scope (e.g., Behrens et al., 2017). Increasing rates of reactive oxygen species (ROS) production at thermal extremes represent another example where increased rates are likely to correspond with decreased performance.

Physiological performance curves can also be exponential, logarithmic, or sigmoidal in shape, although it may be important to restrict our discussion to ecologically relevant conditions. “All things are poison,” as Paraclesus stated, and performance will ultimately suffer when any variable exists in excess. Some physiological rates increase *exponentially* over environmental gradients. The thermal sensitivity of MR in ectotherms, for instance, often follows an exponential curve (see McKenzie, 2011 for review). Increases in free energy drive metabolic reactions forward with temperature coefficients (Q_10_) typically between two and three for biological systems. In other words, physiological reaction rates double to triple for every 10°C increase, and are halved or third for every 10°C decrease (Rogers, 1911). While RMRs ultimately tend to fail with extreme heat, ectotherms typically succumb to thermal stress first. Pcrit curves, on the other hand, tend to be *logarithmic* (e.g., Richards, 2011; Stoffels, 2015), where oxyregulators defend RMR over an impressive range of PO_2_ but ultimately conform to oxygen limitations at some critical PO_2_ threshold (Pcrit). Another common type of performance curve in physiology is dose-response curves that are classically *sigmoidal* in shape (Meddings et al., 1989; but see Calabrese and Baldwin, 1999). Here, increasing doses (i.e., treatment concentration or time) of a drug, toxin, or pollutant may alter performance exponentially across a threshold range, beyond which increases in dose have ever-decreasing effects on performance. Rats treated with methamphetamine, for instance, increase heart rate by approximately 60 beats per minute (bpm) as doses rise between 0.1 and 1mg/kg. Outside this range, however, increases or decreases in dose have diminishing effects on heart rate (Hassan et al., 2016).

### Curve Shifts

#### Adaption

**Figure 3:**
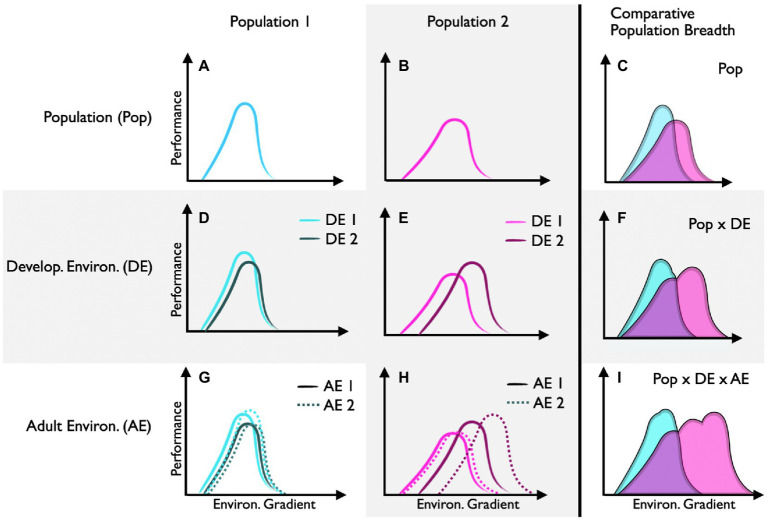
Performance curve shifts between and within populations. Performance curves for population 1 **(A)** and population 2 **(B)** are shifted **(C)**, suggesting that each population is locally adapted (assuming common-garden conditions). Population 1 has low capacity for developmental plasticity **(D)**, whereas population 2 has high capacity for developmental plasticity **(E)**, which increases its potential performance breadth **(F)**. Population 1 has low capacity for reversible acclimation regardless of the developmental environment **(G)**. Population 2 has high capacity for reversible acclimation, but only when individuals develop in environment 2 **(H)**. This relatively high capacity for reversible acclimation further increases the potential performance breadth of population 1 **(I)**.

Performance curves that differ between populations (intraspecific) and species (interspecific) can reflect adaptive differences in environmental tolerance (e.g., Figures 3A–C). This is particularly true when populations are genetically distinct (i.e., minimal geneflow). Many field-based studies show compelling evidence for local adaption. However, without controlled common-garden experiments, local adaption is often difficult to disentangle from the potential effects of developmental plasticity. Likewise, it is often difficult to demonstrate that the trait in question is truly adaptive in animal systems (but see lizard running paper) or that the trait was actively selected for (rather than the consequence of bottleneck events or drift). Despite these distinct challenges, recent work across eight populations of marine snail (*Urosalpinx cinerea*) suggested that shifts in thermal performance curves for growth represented local adaptation to seasonal growth periods (Villeneuve et al., 2021). Specifically, high-latitude populations of snails had higher thermal optima, presumably to achieve large body sizes over a shorter seasonal growth window. Here, common-garden experiments suggest these patterns reflect an adaptive trait (as opposed to developmental plasticity) and insights into the underlying genetic diversity of similar gastropod populations indicate selection (as opposed to a founder event; Villeneuve et al., 2021).

#### Developmental Plasticity

Performance curves that differ as a function of the developmental environment can reflect developmental plasticity (e.g., Figures 3D–F). Here, epigenetic modifiers regulate traits that are generally thought to be irreversible (but see Burggren, 2020) in response to early environmental cues. In this special issue, Rebolledo et al. (2021) show that planktonic survival of developing Australian tubeworms (*Galeolaria caespitosa*) is optimized to the temperatures experienced during embryogenesis. Specifically, there was a warmward skew in thermal survival curves with increasing embryonic temperature. Together, these findings demonstrate that specific aspects of the thermal performance curve (in this case thermal optima, but not thermal limits or thermal breadths) are shaped differently by developmental conditions.

Clonal organisms represent a particularly powerful tool for studies in developmental plasticity because genetic backgrounds are easily controlled. In this special issue, Laskowski et al. (2021) show that the clonal Amazon molly (*Poecilia formosa*) adjusts the thermal sensitivity of open-field swimming behavior, but not maximal swimming performance, as a function of developmental temperature. Interestingly, the authors find the opposite pattern in the closely related but sexually reproducing Atlantic molly (*Poecilia mexicana*), where performance curves for maximal swimming performance, but not open-field swimming behavior, shift as a factor of developmental temperature. This divergence between species suggests that genetic mechanisms are important in mediating the strength of epigenetic responses.

Developmental plasticity can itself represent an adaptive trait. That is, the capacity for developmental plasticity can vary between individuals and populations (e.g., Figures 3D,E). In theory, developmental plasticity is thought to be favored in variable environments where early developmental conditions can project future environments. In this special issue, Smith et al. (2021) found that populations of the grasshopper (*Melanoplus boulderensis*) from higher elevations have greater capacity for developmental plasticity in response to seasonal changes (thermal variability and photoperiod) in the environment. Here, shifts in thermal performance curves for hopping distance and feeding rates revealed that different populations (and sexes) responded to developmental changes in day-length and thermal variability in complex ways. Together this work suggests that the interaction between genotype (populations) and epigenetics (responses to the developmental environment) shape ecologically important responses.

#### Reversible Acclimation

Performance curves that shift temporally within individuals as a function of the environment can reflect acclimation (e.g., Figures 3G–I). While acclimation is a physiological remodeling that is reversible over time, most studies use population-level sampling (as opposed to an individual-level approach) to quantify mean acclimation responses. In other words, studies often acclimate different subsamples of individuals from the same population to different environmental conditions to compare means, as opposed to reversibly acclimating the same individuals (but see x). Often, logistics, life-histories, and the delopmental stages of a focal animal can make experimental designs that acclimate the same individual to multiple environmental conditions challenging if not impossible. In this special issue, for instance, Longhini et al. (2021) analyzed thermal performance curves for aerobic scope in tadpoles of the stream-breeding savanna tree frog (*Bokermannohyla ibitiguara*) to determine their capacity for reversible acclimation. Here, a within-individual approach would likely not be possible because temporal responses to the changing thermal environment would be confounded with developmental stage. While the authors found that thermal performance curves for aerobic scope shifted with acclimation temperature, the effect is likely to reflect a pathology of high acclimation temperature rather than a regulated compensatory response. That is, aerobic scope collapsed in tadpoles acclimated to the upper boundary of temperatures naturally experienced in their microhabitat (i.e., 25°C). Thus, not all curve shifts within and between individuals represent adaptive plastic responses – a comprehensive understanding of the performance parameter in question, the life history of the focal species, and the acute effects of the environmental input(s) used represent important points of interpretation.

An increasing body of work has also revealed that there can be substantial inter-individual variation in the capacity for acclimation; that is, some individuals have phenotypes that are particularly plastic, and others have phenotypes that are more fixed (e.g., x). While some of this variation is likely to be genetically determined, the capacity for reversible acclimation itself also appears to be a plastic trait that can be programmed in response to the developmental environment. An implication of individual variation in the capacity for acclimation is that mean acclimation responses cannot predict population responses to environmental change in meaningful ways. With a bet-hedging strategy, for instance, only a subsection of the population may persist under conditions that favor plastic over fixed phenotypes. In this special issue, Seebacher and Little (2021) collate published data from 608 mosquitofish (*Gambusia holbrooki*) that were each reversibly acclimated to both a cool and warm temperature to show that focusing on mean values can mask underlying variation and obscure bet-hedging dynamics – particularly when populations are undersampled. Further, by focusing on plasticity at an individual level, Seebacher and Little (2021) also investigate tradeoffs (and potential costs) associated with plasticity, including a tradeoff between plasticity and maximal performance.

### Curves Beyond Boundaries

#### Reductionist Mechanisms

Shapes of performance curves across functional boundaries and levels of biological organization can be particularly useful to determine potential mechanisms underlying higher-level traits. In this special issue, Rollwitz and Jastroch (2021) used plate-based respirometry to investigate the mechanisms compromising oxygen consumption rates of zebrafish (*Danio rerio*) embryos at thermal extremes. Specifically, the authors used a targeted pharmacological approach to generate thermal reaction norms for mitochondrial (e.g., basal mitochondrial respiration, ATP-linked respiration, and coupling efficiency) and cardiac (heart rate) performance in embryos pre-exposed to a range of five temperatures. Collectively, their work demonstrates that comparing performance curves down levels of biological organization can help elucidate mechanisms driving whole-animal responses. A note of particular interest is that the mechanisms that modulate mitochondrial performance with changing temperatures are non-linear – mitochondrial oxygen consumption is constrained by reduced rates of ATP production at low temperatures and amplified by increasing rates of proton leak at high temperatures.

#### Emergent Properties

In the same way performance curves can help uncover mechanisms underlying physiological responses to the environment, they can also be used to make predictions about emergent properties of a system at higher levels of organization. In this special issue, Killen et al. (2021) explore how within- and between-individual variation in physiological performance curves can shape social behavior, and higher order interactions like collective movement, disease and parasite transfer, and predator-prey relationships. Specifically, Killen et al. (2021) use thermal performance curves to model how differences in individual responses to temperature (e.g., the rank order of performance capacity as temperatures change) can impact group dynamics. Their work is comprehensive in the sense that they consider how the different parameters that comprise the thermal performance curve (i.e., peak performance, optimal performance, performance breadth, and critical limits) may shift between individuals as environments change. This type of approach is particularly important to link changes in abiotic stressors, which typically act at the molecular-, cell-, or tissue-level, with their environmental, economic, and cultural impacts, which are typically assessed at population-, community-, and ecosystem-levels. While their work here is primarily theoretical, the authors make specific recommendations for empirical studies testing the effects of individual variation in physiological performance curves on social dynamics.

## Conclusion

Performance curves represent a powerful tool to understand the implications of dynamic traits in changing environments. While the approach itself has been around for more than a century, this special issue showcases underused or emerging ways in which performance curves can be applied in comparative fields. We partitioned curve shapes, curve shifts, and functional boundaries into distinct subsections for simplicity, but we acknowledge that biological systems are rarely so defined. The complexity of natural systems is reflected by the studies included in this special issue, where comparisons within and between individuals, populations, endotherms and ectotherms (Levesque et al., 2021), and phylogenetic differences between species highlight the complex interactions that exist between evolutionary forces and epigenetic mechanisms underlying plasticity. Focusing on multiple traits across functional boundaries can also help elucidate mechanisms that underlie animal responses to environmental change and make predictions about the impacts of lower-level responses on higher levels of biological organization. Combined, these approaches are particularly powerful to identify how individual variation in environmental responses might scale up to the population-, species-, and community-level dynamics that inform conservation management strategies.

## Author Contributions

AL and FS conceived the ideas. AL wrote the manuscript. FS edited the manuscript. All authors contributed to the article and approved the submitted version.

## Funding

AL was supported by a Discovery Grant from the Natural Sciences and Engineering Research Council of Canada (NSERC; RGPIN-2021-03142). FS was supported by an Australian Research Council Discovery Grant (DP190101168).

## Conflict of Interest

The authors declare that the research was conducted in the absence of any commercial or financial relationships that could be construed as a potential conflict of interest.

## Publisher’s Note

All claims expressed in this article are solely those of the authors and do not necessarily represent those of their affiliated organizations, or those of the publisher, the editors and the reviewers. Any product that may be evaluated in this article, or claim that may be made by its manufacturer, is not guaranteed or endorsed by the publisher.
